# The Influence of Urotropin on the Urine

**Published:** 1908-10

**Authors:** 


### ABSTRACTS.

---

### The Influence of Urotropin on the Urine.

Interesting findings are reported by Richard Stern (*Zeitschrift f. Hygiene u. Infektionschrank.*, Vol. 39, 1908), in which he says: with urotropin a liberation of formaldehyde or antiseptically-acting formaldehyde compounds best takes place in acid urine. A urotropin urine with alkaline reaction is less antiseptic. By alkaline urines Stern means urines to which sodium bicarbonate has been added. There is therefore no contradiction between his experimental results and the clinical observation that urotropin acts well when the urinary reaction is alkaline. For a clinically alkaline urine means one which has undergone ammoniacal decomposition—one which is acid in the kidneys and is only in the bladder infected with uric acid-decomposing bacteria. For the practical employment of urotropin it is important not to weaken its action by simultaneous free administration of alkalies.

Though free flushing of the urinary organs is rightly regarded as an important therapeutic measure, it involves diluting the antiseptic in the urine. Therefore, when the dose of urotropin can not be proportionately increased, the patient should be ordered to drink large amounts of liquids throughout the day, while larger doses of urotropin are given morning and evening.

Deep seated inflammatory processes of the bladder, renal pelvis, and the like, are less amenable to urinary antiseptics. In tuberculosis and other deeply penetrating infectious processes, a cure can not be attained with urotropin. The remedy is, however, of service by restraining bacterial development in the urine and alleviating the irritating action of the urine on the diseased mucosæ.

Stern thinks urotropin is by no means used enough before and after instrumental procedures on the urinary organs. Urotropin in large doses uniformly distributed (60 grains a day in 3 to 6 doses) should be given in all cases in which obstructed urination favors infection, before and after the introduction of instruments (catheters, cystoscopes), and before and after gynecological or surgical procedures in which injury or contusion of the bladder may occur. Stern recommends a large dose late at night, to render the nocturnal urine antiseptic. Otherwise much of the success attained during the day is negatived during the night. In phosphaturia, says Stern, urotropin acts when the excretion of urine turbid with earthy phosphates is due to ammoniacal fermentation. His cases of this class were mostly of preceding gonorrhea in which secondary staphylococcus infection occurred. In neurasthenics, who often suffer from hyperacidity and constipation and therefore freely take alkaline waters and vegetable acid salts, the food alone may suffice to produce an alimentary phosphaturia. Here urotropin is unavailing. But when the urine of such patients is weakly acid or neutral, a moderate formation of ammonia or uric acid-decomposing organisms suffices to induce precipitation of earthy phosphates. A stronger urinary infection can, of course, also lead to phosphaturia, even if the food is not responsible. In such cases the phosphaturia is rapidly obviated by urotropin in medium-sized doses. Usually the urotropin must be given continuously because generally only an inhibition of uric acid-decomposing organisms is effected.

Stern considers it erroneous to speak of the dose. It varies in accordance with the resistance of the disease producers and other factors in each individual case. Often when 7½ grains urotropin thrice daily proved unavailing, the number of bacteria rapidly decreased when the dose was doubled or trebled.

